# Seafood safety and consumption in coastal Bangladesh: unpacking knowledge, attitudes, preferences, and environmental concerns

**DOI:** 10.1017/jns.2025.25

**Published:** 2025-05-21

**Authors:** Nitai Roy, Sultan Mahmud Imran, Aysha Siddiky, Samia Sultana, Sumana Mahmud, Abdullah Al Adib, Kamal Krishna Biswas

**Affiliations:** 1 Department of Biochemistry and Molecular Biology, Patuakhali Science and Technology University, Patuakhali, Bangladesh; 2 Faculty of Nutrition and Food Science, Patuakhali Science and Technology University, Patuakhali, Bangladesh; 3 Department of Biochemistry and Molecular Biology, University of Rajshahi, Rajshahi, Bangladesh

**Keywords:** Bangladesh, Coastal communities, Consumer behaviour, Food contamination, Food safety, Seafood, GDP, gross domestic product, EPA, eicosapentaenoic acid, DHA, docosahexaenoic acid, NCDs, non-communicable diseases, PCBs, polychlorinated biphenyls, MeHg, methylmercury, CSPI, Centers for Science in the Public Interest, GBM, Ganges Brahmaputra Meghna, NGOs, non-governmental organisations, IUU, Illegal, Unreported and Unregulated

## Abstract

The objective of this study was to explore the knowledge and attitudes regarding seafood safety and consumption, along with preferences and environmental issues in coastal Bangladesh. A cross-sectional, consumer-based survey was conducted in Bangladesh from September to November 2023, targeting 1100 participants aged 18 years and older across seven coastal districts. Convenience sampling and in-person interviews were used for the data collection. The average knowledge and attitude scores toward seafood safety and consumption were 48.2% and 63.5%, respectively. Several factors influenced seafood safety and consumption knowledge, including age, education level, family size, religion, and residence in coastal areas (all P < 0.05). In contrast, attitudes toward seafood safety and consumption were shaped by education level, family size, employment status, seafood allergies, and history of seafood poisoning (all P < 0.05). The most commonly consumed seafood was rupchanda, followed by shrimp. Most participants consumed seafood for its health benefits, with no significant seasonal impact on seafood consumption. Overfishing and climate change were recognised as the most alarming environmental dangers identified by the participants. Coastal communities in Bangladesh have demonstrated moderate attitudes, but relatively low knowledge of seafood safety and consumption. Targeted educational programmes, including community workshops on safe handling and storage, school-based programmes on marine conservation, and digital campaigns via SMS/social media, are needed to improve seafood safety knowledge, while promoting sustainable consumption practices is crucial for addressing environmental concerns like overfishing. Additionally, improving market accessibility and highlighting the health advantages of seafood can drive more informed and healthier consumption choices.

## Introduction

Bangladesh, a South Asian country bordered by the Bay of Bengal, has a coastline of approximately 714 km and a coastal area of 2.30 million hectares.^(1)^ This region, renowned for its rich marine biodiversity, plays a critical role in the livelihood, food security, and cultural identity of millions of people living along the coast.^(2)^ Bangladesh has been ranked among the top five fish-producing countries globally, with its production increasing by 53% since 2009.^(3–5)^ The fisheries sector in Bangladesh not only contributes significantly to the economy but also shapes the dietary practices of coastal communities, where seafood, particularly fish, is a staple. Moreover, following maritime boundary settlements with Myanmar and India, marine capture from the Bay of Bengal is expected to increase substantially, boosting the productivity.^(6,7)^ Currently, seafood is the second most valuable export commodity, contributing 3.7% to Bangladesh’s Gross Domestic Product (GDP), 25.3% to agricultural products, and 2% to total export earnings, valued at $526.45 million USD.^(8–12)^ As the global focus on sustainable fisheries, environmental conservation, and consumer health intensifies, understanding seafood consumption behaviour in Bangladesh has become increasingly vital.^(13)^

Globally seafood is well known for its health benefits. It is a rich source of long-chain omega-3 polyunsaturated fatty acids (LC n-3 PUFAs), including eicosapentaenoic acid (EPA) and docosahexaenoic acid (DHA), which are known to have protective effects against cardiovascular diseases and other non-communicable diseases (NCDs).^(14)^ Additionally, seafood is an excellent source of high-quality proteins, vitamins (e.g. vitamin D), and essential minerals, such as iodine and calcium, while being low in saturated fats.^(15)^ The American Heart Association recommends consuming at least two servings of fish per week, particularly fatty fish, to promote healthy heart.^(15)^ Despite these well-documented health benefits, there are growing concerns about the safety of seafood among consumers, health professionals, and environmental experts owing to the presence of environmental contaminants such as polychlorinated biphenyls (PCBs), dioxin-like compounds, and methylmercury (MeHg). Chronic exposure to these substances can have serious health consequences including neurotoxicity, immunotoxicity, carcinogenicity, and reproductive and developmental issues.^(15)^ This duality, where seafood is both a health-promoting and potentially hazardous food, creates a complex dynamic that influences consumer behaviour, particularly in coastal regions, where seafood is a staple food. However, there remains a significant gap in the understanding of how safety concerns and health benefits affect seafood consumption behaviour Bangladesh’s coastal communities.

Seafood is a popular food category, but it carries a significant risk of diseases transmission. According to data from the U.S. Centers for Science in the Public Interest (CSPI), seafood was linked to 838 outbreaks and 7,298 illnesses between 1998 and 2007.^(16)^ From 2001 to 2010, seafood accounted for 23% of the reported foodborne outbreaks, and between 2011 and 2014, it caused illness in 260,000 Americans.^(17,18)^ Seafood in Bangladesh is a major source of foodborne diseases, with pathogens such as *Salmonella typhi*, *Vibrio cholerae*, *Vibrio parahaemolyticus*, and *Vibrio vulnificus* contributing to outbreaks. These pathogens thrive in unsanitary seafood processing environments and can be exacerbated by consumption of raw or undercooked seafood among local populations.^(19,20)^ Local studies have reported high contamination levels, particularly in urban markets, where Vibrio species are frequently detected.^(19,20)^ Moreover, the nature of seafood supply chains in Bangladesh, marked by low regulatory control, can cause Vibrio spp. and other foodborne diseases to proliferate throughout the distribution process, thereby increasing the risk of outbreaks.^(21,22)^ However, comprehensive studies on the occurrence and outbreaks of foodborne diseases related to seafood are lacking, making it difficult to assess the true scope of safety issues in coastal communities.

In Bangladesh, seafood consumption contributes significantly to the national diet by providing 63% of the total dietary protein.^(23)^ However, this critical food resource is currently threatened. Overfishing, habitat destruction, and climate change have led to marked declines in the quality and quantity of marine resources, with the proportion of coastal fish stocks declining from 17.7% to 14.7%.^(24)^ These environmental challenges raise concerns about the sustainability of fisheries as well as the safety of seafood consumed by coastal populations. Furthermore, as marine pollution increases, the risk of contamination increases, further jeopardising the health of communities dependent on seafood as their primary protein source.

Despite the central role seafood in the diet of coastal Bangladesh, there has been limited research on their knowledge and attitudes towards seafood safety.^(25)^ There is a significant gap in understanding how coastal communities perceive the risks associated with seafood consumption and how these perceptions shape their behaviour. Consumer knowledge of foodborne pathogens, contamination risks, and preventive measures is essential for promoting seafood safety; however, many individuals remain uninformed about these dangers.^(26)^ This knowledge gap is further exacerbated by socio-economic and educational disparities, which may result in differences in risk perception and seafood consumption behaviour among various demographics. Additionally, previous studies have primarily concentrated on the production and commerce of seafood, with limited emphasis on consumer-side perspectives, particularly in vulnerable coastal populations.

The current study aimed to address these gaps by exploring the knowledge and attitudes related to seafood safety and consumption among coastal populations in Bangladesh. Specifically, this research aims to: (i) assess the knowledge and attitudes of consumers regarding seafood safety and consumption, (ii) identify the factors influencing these behaviours, (iii) examine the motivators and demotivators in seafood purchasing decisions, and (iv) explore consumer awareness of environmental issues related to seafood. By gaining a deeper understanding of these aims, this study will provide valuable insights to identify misconceptions and demographic factors that shape seafood consumption, and develop strategies to promote informed and safe seafood consumption. It also highlights the need for policies that balance food security, environmental conservation, and public health, and inform targeted educational interventions to address existing gaps.

## Methodology

### Study settings

**Fig. 1.**
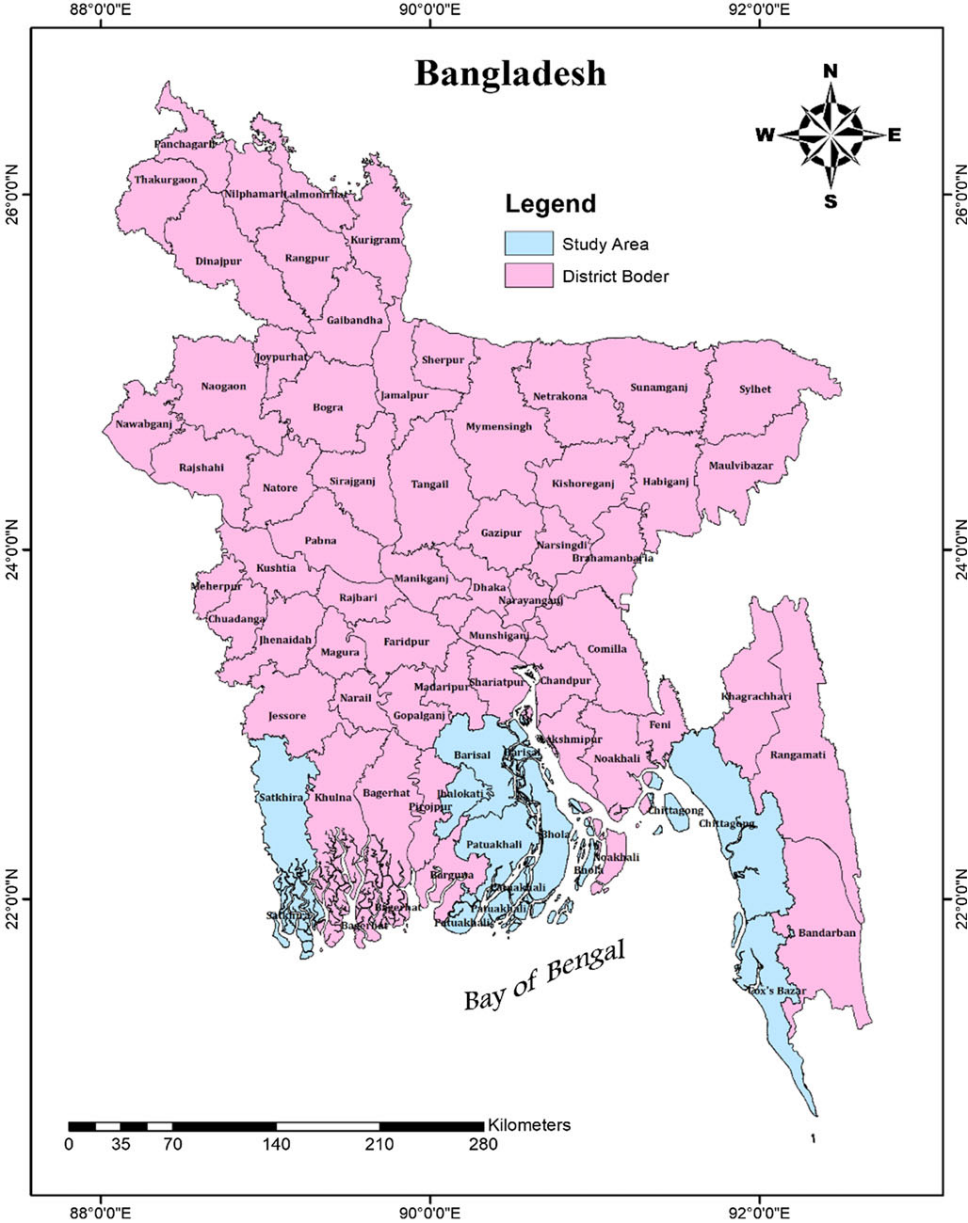
Study area.

The coastal zone of Bangladesh encompasses 19 districts: Jessore, Narail, Gopalganj, Shariatpur, Chandpur, Satkhira, Khulna, Bagerhat, Pirojpur, Jhalakati, Barguna, Barisal, Patuakhali, Bhola, Lakshmipur, Noakhali, Feni, Chittagong, and Cox’s Bazar.^(27)^ This region is geomorphologically and hydrologically shaped by the Ganges Brahmaputra Meghna (GBM) river system and the Bay of Bengal. The coastal zone covers an area of 47,201 km^2^, accounting for 32% of the country’s total land area. Approximately, 35 million people (29% of the national population), live in this zone.^(27)^ This study was conducted among consumers from seven randomly selected coastal districts: Barisal, Bhola, Chittagong, Cox’s Bazar, Jhalakati, Patuakhali, and Pirojpur (See Fig. 1).

### Study design and sampling

A consumer-based, cross-sectional study was carried out between September 1, 2023, and November 30, 2023 to assess consumers’ knowledge, attitudes, and behaviours regarding seafood consumption in the coastal areas of Bangladesh. Participants were included if they met the following criteria: (i) aged 18 years or older and (ii) Bangladeshi by birth. Individuals who were unwilling to participate or had mental health conditions that impeded their ability to provide informed responses were excluded. Participants were recruited using convenience sampling, and data were collected from easily accessible consumers in randomly selected coastal districts. The use of convenience sampling in this study was based on the need to gather data efficiently within a specific timeframe. The convenience sampling used in this study allows for the inclusion of participants who are easily accessible, making it a practical approach given the geographical constraints and objectives of the study. This study adhered to the ethical principles outlined in the Declaration of Helsinki, ensuring that all procedures involving human participants were conducted in accordance with its guidelines.^(28)^

### Sample size calculation

The sample size was calculated using Cochran’s formula, n_o_ = Z^2^pq /e2, where n_o_ represents the estimated sample size, Z is 1.96 at a 95% confidence interval, e is the margin of error set at 5% and q = 1–p. Given the absence of prior studies on consumers’ knowledge, attitude, and behaviour about seafood in the coastal areas of Bangladesh, a conservative estimate of P = 0.5 was used. Using this formula, a minimum sample size of 384 was calculated. However, to account for potential non-responses and missing data, a larger sample of participants was used. After excluding inconsistent or incomplete responses, a final sample of 1100 participants were included in the analysis.

### Interviews and data collection

Data were collected through face-to-face interviews, using a structured questionnaire. The questionnaire was developed based on a comprehensive review of the relevant literature.^(29–31)^ The questionnaire was pilot tested with a convenience sample of 10 consumers and necessary modifications were made based on the feedback received. Seven interviewers, each assigned to a distinct district, collected the data. Prior to the survey, the lead investigator conducted an intensive training session covering the content of the questionnaire, interview techniques, and the study’s inclusion and exclusion criteria. The interviews were scheduled at times convenient for the participants and took place in diverse locations, including local markets, shopping centres, community gathering points, private households, and other public spaces. The trained data collectors first briefed the participants on the study objectives and sought voluntary participation. Written consent was obtained from those who agreed to participate and verbal consent was obtained from illiterate participants. The duration of each interview was approximately 12–15 minutes.

### Study variables and measures

#### Sociodemographic factors

The sociodemographic information collected included various variables, including gender, age, educational qualification, religion, residential status, employment status, total family members, living in coastal areas, statement of food allergy, and history of food poisoning. Family monthly income was categorised into predefined ranges (less than 15,000 BDT/USD 125, 15,000–30,000 BDT/USD 125–250, and more than 30,000 BDT/USD 250. Seafood allergy and history of seafood poisoning were self-reported based on the participants’ personal experiences rather than medical diagnoses.

#### Consumers knowledge towards seafood safety and consumption

To evaluate consumer knowledge regarding seafood consumption in coastal areas, a structured set of 13 close-ended questions was administered, with three response options: ‘True’, ‘False’, and ‘Don’t know’. Each correct response of ‘True’ was awarded 1 point, while incorrect answers marked as ‘False’ or “’Don’t know’ were assigned 0 points. However, for specific questions (i.e. 3, 8, 9, and 12), a ‘False’ response was deemed correct and thus scored 1 point, whereas ‘True’ and ‘Don’t know’ responses were given 0 points.

#### Consumers’ attitude towards seafood safety and consumption

Participants’ attitudes toward seafood consumption were assessed using 18 statements. These statements focus on issues such as trust, environmental concerns, purchasing decisions, and health maintenance. All statements had five possible answers: ‘Strongly Disagree’, ‘Disagree’, ‘Neither Agree nor Disagree’,’ ‘Agree’, and ‘Strongly agree’. Each response was scored accordingly: ‘Strongly Disagree’ received 1 point, ‘Disagree’ 2 points, ‘Neither Agree nor Disagree’ 3 points, ‘Agree’ 4 points, and ‘Strongly agree’ 5 points.

#### Motivators, demotivators, environmental concerns, and consumer preferences

The participants were asked to identify the factors influencing their seafood purchasing decisions. Motivators assessed freshness, safety, health benefits, taste, sustainable sourcing, and ease of preparation, while demotivators such as dislike seafood, lack of cooking knowledge, high cost, and religious restrictions were also recorded. The environmental concerns explored include overfishing, climate change, pollution, species loss, and illegal fishing. Furthermore, consumer preferences are assessed in relation to health advantages, local sourcing, preparation techniques, frequency of consumption, and information sources (markets and personal networks).

### Statistical analyses

Data analysis was conducted using SPSS version 27.0. Descriptive statistics, including frequencies, percentages, means, and standard deviations were calculated. The Shapiro–Wilk test was employed to assess data normality, with a P-value > 0.05 indicating normally distributed continuous variables. Due to the non-normal distribution of data, the Mann–Whitney *U* test and Kruskal–Wallis test were applied to compare the mean scores of knowledge and attitudes towards seafood consumption. Specifically, the Mann–Whitney *U* test was used for variables with two groups (e.g. gender), whereas the Kruskal–Wallis test was employed for variables with more than two groups (e.g. education level). Data transformation was not attempted because nonparametric tests are appropriate for analysing ordinal or skewed data without assuming normality. Linear regression was performed to determine the factors associated with the knowledge and attitude scores. The internal consistency of the knowledge and attitude items was evaluated using Cronbach’s alpha, yielding coefficients of 0.736 for knowledge and 0.843 for attitude.

## Results

### Socio-demographic characteristics of seafood consumers

The socio-demographic characteristics of the participants are summarised in Table 1. Of the 1,100 seafood consumers surveyed (mean age 37.74 ± 13.63), 62.9% were male and 31.5% were between the ages of 26 and 35. More than half (55.7%) had attained an education level of honours or above. A majority (67.6%) resided in rural areas, and 68.5% were unemployed (the unemployment rate reflects formal employment status and does not account for informal or irregular employment). Regarding income, 45.2% reported a monthly household income of 15,000–30,000 Bangladeshi Taka. Additionally, 63.5% of the participants came from families with 4–6 members, 42.2% lived in coastal areas, 77.2% reported no seafood allergies, and 86.9% had no history of seafood poisoning. The gender distribution imbalance, with a higher proportion of male participants, may be attributed to cultural and societal factors (including traditional gender roles, occupational differences, and survey participation tendency) that influence seafood purchasing and consumption behaviours.

**Table 1.** Socio-demographic characteristics of seafood consumer (N = 1100)

Variables	Frequency	Percentage (%)
**Gender**		
Female	408	37.1%
Male	692	62.9%
**Age of the participant (mean age 37.74 ± 13.63)**		
18–25	222	20.2%
26–35	346	31.5%
36–45	273	24.8%
46–55	97	8.8%
56–65	109	9.9%
65+	53	4.8%
**Educational Qualification**		
No Formal Education	27	2.5%
Primary	115	10.5%
Secondary	136	12.4%
Higher Secondary	209	19.0%
Honours or above	613	55.7%
**Religion**		
Islam	880	80.0%
Hindu	180	16.4%
**Others^a^**	40	3.6%
**Residential status**		
Rural	744	67.6%
Urban	356	32.4%
**Employment status**		
Unemployed	754	68.5%
Employed	346	31.5%
**Total family members**		
1–3	286	26.0%
4–6	699	63.5%
Above 6	115	10.5%
**Family monthly income (BDT)**		
<15000	408	37.1%
15000–30000	497	45.2%
>30000	195	17.7%
**Living in Costal area**		
No	636	57.8%
Yes	464	42.2%
**Statement of Sea food allergy**		
No	849	77.2%
Yes	251	22.8%
**History of sea food poisoning**		
No	956	86.9%
Yes	144	13.1%

a Included Buddhism and Christianity.

### Evaluation of seafood safety and consumption knowledge

As shown in Table 2, the respondents displayed varying levels of knowledge of seafood safety and consumption, with an overall correct response rate of 48.2%. Most respondents (60.8%) were aware that Hilsa fish are rich in omega-3 fatty acids, and 43.6% knew that the recommended intake of seafood was twice a week. However, only 23.3% believed that frozen seafood is nutritionally superior to fresh seafood, while 62.7% recognised the threat of overfishing. Regarding health, 43.1% correctly identified oily fish as a source of omega-3s, 58.1% linked crab and shrimp to high cholesterol levels, and 71.4% understood the high protein content of seafood. However, only 29.4% had a broader understanding of ‘sustainable seafood’ beyond fishing practices, 47.2% were unsure about microplastic contamination, 68.2% recognised the risks of mercury, and 57.3% acknowledged bacterial contamination as a concern. Significant variations in the mean score for seafood consumption knowledge were identified across different sociodemographic factors such as age, educational qualification, religion, place of residence, total family members, family monthly income and living in coastal communities (Table 4).

**Table 2.** Consumers knowledge about seafood safety and consumption

Knowledge statements (α = 0.736)	Mean ± SD^b^	True (%)	False (%)	Don’t know (%)
Hilsa fish is rich in omega-3 fatty acids, contributing to its reputation as a healthy seafood choice	0.61 ± 0.49	669 (60.8)	46 (4.2)	385 (35.0)
Health organisations recommend eating seafood twice a week	0.44 ± 0.50	480 (43.6)	117 (10.6)	503 (45.7)
**Frozen seafood has higher nutritional value than raw seafood^a^**	0.46 ± 0.50	256 (23.3)	504 (45.8)	340 (30.9)
As a major global concern, overfishing threatens the availability of many seafood species	0.63 ± 0.48	690 (62.7)	139 (12.6)	271 (24.6)
Oily fish, like salmon and mackerel provide heart-healthy omega-3 fatty acids	0.43 ± 0.50	474 (43.1)	137 (12.5)	489 (44.5)
Consuming certain seafood, such as crab and shrimp, can contribute to high cholesterol levels	0.58 ± 0.50	639 (58.1)	112 (10.2)	349 (31.7)
High-quality protein is abundant in seafood	0.71 ± 0.45	785 (71.4)	86 (7.8)	229 (20.8)
**The term ‘sustainable seafood’ solely relates to the method of fishing, not the total influence on the marine environment^a^**	0.24 ± 0.43	323 (29.4)	262 (23.8)	515 (46.8)
**Microplastic contamination of Bangladeshi seafood does not appear to be a significant issue^a^**	0.27 ± 0.45	283 (25.7)	298 (27.1)	519 (47.2)
Consuming too much mercury, which is present in several sea fish species, may be harmful to human health	0.68 ± 0.47	750 (68.2)	95 (8.6)	255 (23.2)
Bacterial contamination is a significant issue in seafood from the coastal areas of Bangladesh, impacting food safety	0.57 ± 0.50	630 (57.3)	150 (13.6)	320 (29.1)
**Allergies to seafood are prevalent, and those who are allergic to a particular variety are probably also allergic to others^a^**	0.20 ± 0.40	429 (39.0)	215 (19.5)	456 (41.5)
The government of Bangladesh has implemented regulations to promote sustainable practices in the country’s seafood industry	0.46 ± 0.50	506 (46.0)	110 (10.0)	484 (44.0)
**Mean score ± Standard Deviation**	**6.27 ± 2.85**			

a These statements were reverse coded.

b Mean score was calculated by dividing the number of correct responses by the total number of responses.

Table 5 presents the results of the linear regression analysis of the factors associated with seafood consumption knowledge. Participants with no formal education (β = –0.12), primary education (β = –0.30), secondary education (β = –0.18), and higher secondary education (β = –0.10), presented a lower level of seafood consumption knowledge than those with honours or above. Participants from families with 4–6 members had higher knowledge levels (β = 0.09) than those from families with more than six members. Additionally, participants aged 56–65 exhibited lower levels of seafood consumption knowledge (β = –0.01) compared to those aged over 65 years. Hindu participants demonstrated higher knowledge levels (β = 0.18) than did Christian and Buddhist participants. Furthermore, participants who did not live in coastal areas exhibited lower levels of seafood consumption knowledge (β = –0.17) than those who lived in coastal areas. All reported associations were considered statistically significant at P < 0.05.

### Evaluation of seafood safety and consumption attitudes

Table 3 reports respondents’ views on trust, environmental, purchasing, and health issues related to seafood. The overall correct response rate for the attitude test was 63.46%. The results indicate that trust in seafood sellers is primarily driven by freshness and quality, with 45.3% of respondents agreeing on its importance, while only 35.1% viewed clear labelling as essential for building trust. Government-backed safety campaigns were widely supported (36.2% agreed, 28.7% strongly agreed). Environmental concerns included reducing overfishing (36.3% agreed) and minimising plastic waste (33.3% agreed) though fewer emphasised local sourcing. Purchasing behaviour showed a moderate interest in sustainable sources (35.4% agreed) and seasonal availability, with Affordability a concern (only 26.5% agreed seafood is affordable). Health perception highlighted seafood’s role in overall well-being (43.0% agreed), and chronic diseases prevention (36.0% agreed), though fewer respondents associated it with weight management.

**Table 3.** Consumers attitudes towards seafood safety and consumption

Attitudes about seafood safety and consumption (α = 0.843)	Mean ± SD^a^	Strongly Disagree (%)	Disagree (%)	Neutral (%)	Agree (%)	Strongly agree (%)
**Trust statements**						
Trusting social media for seafood info is useful.	3.16 ± 1.10	112 (10.2)	140 (12.7)	415 (37.7)	321 (29.2)	112 (10.2)
Freshness and quality are key factors in trusting seafood sellers.	3.64 ± 1.03	43 (3.9)	118 (10.7)	234 (21.3)	498 (45.3)	207 (18.8)
Selecting trustworthy sellers is crucial for my seafood choices	3.71 ± 1.04	58 (5.3)	68 (6.2)	257 (23.4)	473 (43.0)	244 (22.2)
Clear labeling on seafood packaging is essential for building trust.	3.49 ± 1.01	38 (3.5)	130 (11.8)	367 (33.4)	386 (35.1)	179 (16.3)
Government-backed safety campaigns is crucial to enhance trust in the seafood industry.	3.78 ± 1.08	52 (4.7)	72 (6.5)	262 (23.8)	398 (36.2)	316 (28.7)
**Environmental statements**						
Contaminants such as mercury in certain types of seafood is bad for pregnant women and young children.	3.63 ± 1.09	57 (5.2)	58 (7.7)	332 (30.2)	360 (32.7)	266 (24.2)
Reducing overfishing is crucial for preserving the balance of marine ecosystems	3.77 ± 1.04	36 (3.3)	85 (7.7)	279 (25.4)	399 (36.3)	301 (27.4)
Minimising plastic waste from seafood packaging is essential for ocean health	3.74 ± 1.04	39 (3.5)	74 (6.7)	322 (29.3)	366 (33.3)	299 (27.2)
Opting for locally sourced seafood reduces the environmental footprint of transportation	3.46 ± 0.95	41 (3.7)	93 (8.5)	439 (39.9)	378 (34.4)	149 (13.5)
Limiting pesticide use in seafood production significantly benefits ecosystems.	3.71 ± 1.13	68 (6.2)	79 (7.2)	268 (24.4)	376 (34.2)	309 (28.1)
**Purchasing statements**						
Purchasing seafood from sustainable sources is significant to me	3.50 ± 0.98	44 (4.0)	92 (8.4)	407 (37.0)	389 (35.4)	168 (15.3)
My seafood choices significantly based on seasonal availability	3.27 ± 0.99	58 (5.3)	146 (13.3)	451 (41.0)	333 (30.3)	112 (10.2)
Seafood is affordable	3.13 ± 1.04	93 (8.5)	159 (14.5)	457 (41.5)	292 (26.5)	99 (9.0)
The origin of seafood influences my purchasing decisions	3.43 ± 0.99	51 (4.6)	109 (9.9)	399 (36.3)	403 (36.6)	138 (12.5)
Seafood freshness significantly influences my purchasing decisions.	3.64 ± 0.98	38 (3.5)	78 (7.1)	336 (30.5)	438 (39.8)	210 (19.1)
**Health statements**						
Seafood consumption is a crucial part of my overall well-being	3.70 ± 1.00	42 (3.8)	79 (7.2)	278 (25.3)	473 (43.0)	228 (20.7)
Seafood consumption lowers the risk of chronic diseases, including cardiovascular diseases	3.73 ± 0.98	30 (2.7)	66 (6.0)	340 (30.9)	396 (36.0)	268 (24.4)
Seafood is an important component of my strategy for weight management	3.22 ± 1.06	93 (8.5)	121 (11.0)	466 (42.4)	295 (26.8)	125 (11.4)
**Mean score ± Standard Deviation**	**63.69 ± 9.70**					

a For each question, we calculate the mean value by dividing the total sum of response scores by the number of respondents who answered that question.

The mean score of attitudes toward seafood safety and consumption varied significantly across several sociodemographic factors, including age, educational qualification, religion, employment status, total number of family members, living in a coastal community, presence of seafood allergies, and experience with seafood poisoning (Table 4).

**Table 4.** Mean score of seafood safety and consumption knowledge and attitudes by demographic characteristics

	Variables	Knowledge		Attitude	
		Mean ± SD	P value	Mean ± SD	P value
**Gender**					
	Female	6.18 ± 3.03	0.480^a^	64.38 ± 8.71	0.067^a^
	Male	6.33 ± 2.75		63.28 ± 10.23	
**Age of the participant**					
	18–25	6.75 ± 2.81	**<0.001**	64.72 ± 9.09	**0.009**
	26–35	5.84 ± 2.55		63.89 ± 9.42	
	36–45	6.52 ± 2.82		62.48 ± 10.49	
	46–55	6.66 ± 2.52		62.55 ± 9.92	
	56–65	5.56 ± 3.50		63.77 ± 9.47	
	65+	6.64 ± 3.56		66.11 ± 9.19	
**Educational qualification**					
	No Formal Education	4.48 ± 3.04	**<0.001**	60.19 ± 9.86	**0.002**
	Primary	3.87 ± 3.09		61.97 ± 7.79	
	Secondary	5.31 ± 3.03		64.79 ± 8.42	
	Higher Secondary	6.19 ± 2.72		64.80 ± 8.45	
	Honours or above	7.05 ± 2.42		63.54 ± 10.57	
**Religion**					
	Islam	6.17 ± 2.89	**<0.001**	63.24 ± 9.92	**0.014**
	Hindu	6.94 ± 2.74		65.43 ± 8.89	
	Other’s	5.53 ± 2.05		65.52 ± 6.80	
**Residence status**					
	Rural	5.98 ± 2.91	**<0.001** **^a^**	63.52 ± 9.76	0.304^a^
	Urban	6.89 ± 2.63		64.03 ± 9.58	
**Employment status**					
	Unemployed	6.38 ± 2.94	0.050^a^	62.76 ± 10.05	**<0.001** **^a^**
	Employed	6.05 ± 2.65		65.70 ± 8.57	
**Total family members**					
	1–3	6.08 ± 2.61	**0.043**	64.83 ± 8.86	**<0.001**
	4–6	6.42 ± 2.91		63.73 ± 9.76	
	Above 6	5.88 ± 3.05		60.59 ± 10.72	
**Family monthly income (BDT)**					
	<15000	5.90 ± 2.79	**<0.001**	63.85 ± 8.72	0.871
	15000–30000	6.36 ± 2.79		63.56 ± 10.35	
	>30000	6.83 ± 3.06		63.67 ± 9.98	
**Living in a coastal community**					
	No	5.89 ± 3.02	**<0.001** **^a^**	63.11 ± 9.67	**0.006** **^a^**
	Yes	6.80 ± 2.51		64.47 ± 9.69	
**Having any sea food allergies**					
	No	6.34 ± 2.90	0.159^a^	64.29 ± 9.56	**<0.001** **^a^**
	Yes	6.06 ± 2.69		61.63 ± 9.89	
**Suffering from seafood poisoning**					
	No	6.27 ± 2.94	0.950^a^	64.16 ± 9.63	**<0.001** **^a^**
	Yes	6.32 ± 2.25		60.54 ± 9.59	
	**Total**	**6.27 ± 2.85**		**63.69 ± 9.70**	

a Mann-Whitney *U* test was conducted, unless otherwise stated = Kruskal Wallis H test was conducted. Bold values indicate statistical significance at *P* <0.05.

Linear regression analysis indicated that participants with secondary (β = 0.07) and higher secondary education (β = 0.07) demonstrated significantly more positive attitudes toward seafood consumption than those with honours or higher-level education. Unemployed participants exhibited significantly lower (β= –0.14) positive attitudes than employed participants. Additionally, participants with family sizes of 1–3 members (β = 0.10) and 4–6 members (β = 0.09) reported more positive attitudes than those with more than six family members. Furthermore, individuals without any seafood allergies were found to have significantly more positive attitudes (β = 0.08) toward seafood consumption than those with allergies. Finally, participants who had not experienced seafood poisoning exhibited more positive attitudes (β = 0.09) than those who had suffered from seafood poisoning (Table 5). All reported associations were considered statistically significant at P < 0.05.

**Table 5.** Association between socio-demographic characteristics and participant knowledge and attitudes about seafood safety and consumption

Variables	Knowledge^b^				Attitude^c^			
	β	95% CI		*P-value*	β	95% CI		*P-value*
		LL	UL			LL	UL	
**Gender**								
Female	0.01	–0.28	0.38	0.761	0.05	–0.09	2.24	0.071
Male	**Reference**				**Reference**			
**Age of the participant**								
18–25	–0.03	–1.04	0.58	0.575	–0.01	–3.18	2.49	0.813
26–35	–0.11	–1.49	0.08	0.079	–0.06	–4.12	1.39	0.331
36–45	–0.07	–1.26	0.35	0.267	–0.08	–4.72	0.94	0.190
46–55	–0.02	–1.18	0.73	0.643	–0.05	–5.02	1.66	0.324
56–65	–0.01	–1.89	–0.06	**0.036**	–0.03	–4.30	2.14	0.510
65+	**Reference**				**Reference**			
**Educational Qualification**								
No Formal Education	–0.12	–3.28	–1.23	**<0.001**	–0.01	–4.45	2.79	0.651
Primary	–0.30	–3.36	–2.24	**<0.001**	0.01	–1.50	2.59	0.602
Secondary	–0.18	–2.08	–1.07	**<0.001**	0.07	0.29	3.89	**0.023**
Higher Secondary	–0.10	–1.16	–0.32	**0.001**	0.07	0.25	3.19	**0.022**
Honours or above	**Reference**				**Reference**			
**Religion**								
Islam	0.10	–0.12	1.61	0.090	–0.02	–3.55	2.53	0.744
Hindu	0.18	0.46	2.29	**0.003**	0.02	–2.76	3.69	0.779
Others^a^	**Reference**				**Reference**			
**Residential status**								
Rural	–0.06	–0.72	0.02	0.064	–0.02	–1.72	0.87	0.517
Urban	**Reference**				**Reference**			
**Employment status**								
Unemployed	0.05	–0.08	0.66	0.120	–0.14	–4.23	–1.63	**<0.001**
Employed	**Reference**				**Reference**			
**Total family members**								
1–3	0.07	–0.12	1.06	0.116	0.10	0.20	4.35	**0.031**
4–5	0.09	0.01	1.07	**0.045**	0.09	0.04	3.76	**0.045**
Above 6	**Reference**				**Reference**			
**Family monthly income (BDT)**								
<15000	–0.04	–0.76	0.32	0.424	0.06	–0.66	3.15	0.200
15000–30000	–0.07	–0.89	0.10	0.120	0.03	–1.18	2.30	0.531
>30000	**Reference**				**Reference**			
**Living in Coastal area**								
No	–0.17	–1.30	–0.62	**<0.001**	–0.03	–1.86	0.54	0.283
Yes	**Reference**				**Reference**			
**Statement of Seafood allergy**								
No	0.05	–0.05	0.77	0.087	0.08	0.50	3.37	**0.008**
Yes	**Reference**				**Reference**			
**History of sea food poisoning**								
No	0.03	–0.27	0.83	0.324	0.09	0.62	4.50	**0.010**
Yes	**Reference**				**Reference**			
**Knowledge**	NI				0.26	0.69	1.10	**<0.001**

*Note*: CI, confidence interval; LL, Lower limit; UL, Upper limit; NI, not indicate.

The bolded values indicate statistical significance at P < 0.05 level.

a Included Buddhism, Christianity.

b Adjusted R square 0.175.

c Adjusted R square 0.120.

### Seafood consumption: motivators barriers, environmental concerns, and consumer behaviour

The results indicate that freshness (16.8%), taste (15.5%), and health benefits (12.7%) were the primary motivators for seafood consumption, followed by safety (11.5%) and price (6.0%) also being influential (Table 6). Factors such as sustainability (4.9%) and local sourcing (6.2%) were mentioned less frequently, while ease of cooking (5.6%) and visual appeal (4.5%) played minor roles. In contrast, major demotivators included the unavailability of local seafood (22.4%), high costs (21.3%), and uncertainty regarding sustainable options (19.9%).

**Table 6.** Motivators and demotivators when purchasing seafood

Items	Frequency	Percentage (%)
Motivators^a^		
Fresh	832	16.8%
Safe to eat	568	11.5%
Good for health/family	630	12.7%
Taste	766	15.5%
Sustainably sourced/environmentally friendly	243	4.9%
Non-GMO	131	2.6%
Price	298	6.0%
A type of food that I have always eaten	110	2.2%
Easy to cook	275	5.6%
Sourced locally	306	6.2%
Visual appeal	224	4.5%
Recipes and cooking tips	259	5.2%
Informational workshops on seafoods	167	3.4%
Social norms and obligations	79	1.6%
Don’t know/unsure	65	1.3%
**Demotivators** ^a^		
I don’t like to eat any seafood	286	11.7%
I don’t know how to cook seafood	289	11.8%
I don’t have access to a kitchen to cook seafood	195	7.9%
I am not sure which types of local seafood are sustainable	489	19.9%
Local seafood is not available where I typically shop for groceries	549	22.4%
Local seafood is too expensive	523	21.3%
Religion	122	5.0%

a Participants were instructed to check all options that apply.

The most alarming environmental concern was overfishing (18.5%), followed by climate change (13.7%) and pollution affecting rivers and streams (13.1%). Participants also expressed significant concern regarding biodiversity loss (11.0%), species decline (9.2%), and illegal fishing activities (8.4%) (Table 7).

**Table 7.** Most alarming environmental dangers seafood consumers aware of

Alarming environmental dangers^a^	Frequency	Percentage (%)
Overfishing	849	18.5%
Climate change	625	13.7%
Pollution & waste damaging rivers & streams	599	13.1%
Extreme weather events/ changing weather patterns	331	7.2%
Air pollution	295	6.4%
Loss/destruction of rain forests	281	6.1%
Loss of animal species	421	9.2%
Loss of wilderness/ urban sprawl	142	3.1%
Loss of biodiversity	504	11.0%
Illegal, Unreported, and Unregulated (IUU) Fishing	383	8.4%
Genetic modification	147	3.2%

a Participants were instructed to check all options that apply.

**Fig. 2.**
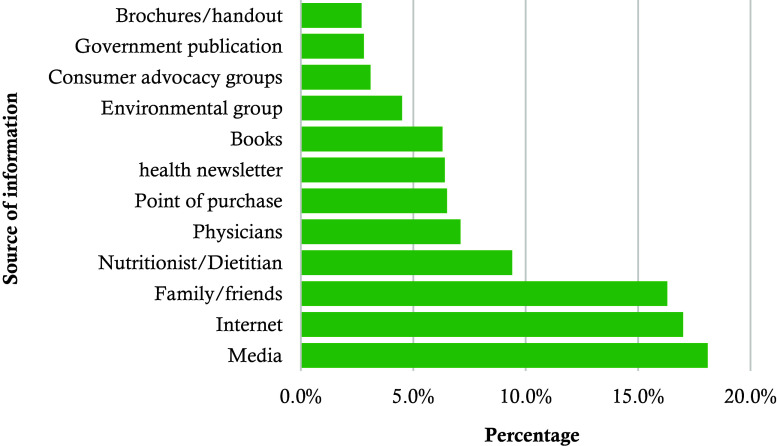
Source of information of consumers for seafood consumption.

Table 8 details consumer preferences and habits related to seafood consumption. Health benefits were the primary reason for consuming seafood, reported by 50.7% of participants, followed by taste (39.5%) and economic considerations (9.7%). The majority of consumers purchased seafood from local fish markets (40.6%), with notable purchases from direct fisherman (22.6%) and Arat/paikar markets (22.5%). When buying seafood, common inquiries included species name (27.8%) and catch date (23.6%), while 10.8% did not ask any questions. Traditional cooking methods were preferred by 56.1% of respondents, and 27.5% favoured frying. Seasonal factors were less significant for 62.8% of consumers, though 28.8% preferred seafood in winter. Regarding frequency, 21.2% consumed seafood twice a week, and 20.8% a few times per month. Among the consumers, 18.1% relied on media as their primary source of information about seafood, while 17% used the internet (Fig. 2). In terms of commonly consumed seafood, rupchanda was the most popular (19.1%), followed by shrimp (18.4%), coral fish (11.5%), and laitta (10.9%) (Table 8).

**Table 8.** Preferences and habits of consumers for seafood consumption

Questions	Frequency	Percentage (%)
**What is the primary reason for seafood consumption?**		
Economic	107	9.7%
Healthy	558	50.7%
Tasty	435	39.5%
**Where do you usually prefer to purchase seafood for consumption?^a^**		
Local fish market	737	40.6%
Arat/ paikar market	408	22.5%
Super shop/ online shop	128	7.1%
Directly from fisherman	410	22.6%
Restaurant	132	7.3%
**When you purchase seafood, which of the following questions do you ask?^a^**		
Species Name	619	27.8%
When did you catch the seafood?	525	23.6%
How did you preserve the seafood?	502	22.6%
Is the seafood wild or farmed?	337	15.2%
I do not ask questions about my seafood.	241	10.8%
**What’s your favourite way to prepare seafood?**		
Grilling	180	16.4%
Frying	303	27.5%
Traditional Cooking	617	56.1%
**Which season do you favour for seafood consumption?**		
Summer	92	8.4%
Winter	317	28.8%
Season has no impact on consumption	691	62.8%
**How often do you consume seafood in your home?**		
Once a week	198	18.0%
Twice a week	233	21.2%
More than twice a week	147	13.4%
Once per month	64	5.8%
A few times per month	229	20.8%
Once every 2–3 Months	128	11.6%
More than once a year	101	9.2%
**What types of seafood do you typically eat?^a^**		
Rupchanda	776	19.1%
Squid	115	2.8%
Coral	469	11.5%
Crab	354	8.7%
Shrimp	747	18.4%
Lobster	124	3.1%
Tuna	280	6.9%
Laitta	444	10.9%
Octopus	92	2.3%
Lakkha	240	5.9%
Others	424	10.4%

a Participants were instructed to check all options that apply.

## Discussion

This study is the first to comprehensively investigate seafood consumption knowledge, attitudes, preferences, and environmental concerns among the coastal residents of Bangladesh. Our findings revealed a notable knowledge deficit among participants, with an overall knowledge score of 48.2%, despite demonstrating moderately positive attitudes (63.5%) towards seafood consumption. Similar patterns have been observed among Brazilian consumers who exhibit lower knowledge and attitudes toward seafood safety.^(29,30)^ Additionally, a study from the United States reported limited knowledge and attitudes of consumers toward seafood safety and seafood consumption.^(32)^ This finding highlights a significant gap between what people know about seafood and how they feel about it. This means that while people may have positive feelings toward eating seafood, such as recognising its health benefits or enjoying its taste, their actual knowledge of important aspects, such as nutritional value, recommended intake, or environmental concern, is limited. This underscores the urgent need for targeted education and public health campaigns to bridge the knowledge gap for informed decision-making and promote sustainable seafood consumption practices. To bridge this gap, multi-pronged educational initiatives should be implemented through: (1) community workshops and cooking demonstrations to teach safe handling practices; (2) integration of seafood nutrition and sustainability topics into school curricula; (3) social media campaigns tailored to different demographic groups; and (4) nationwide public awareness programmes. The Department of Fisheries and the Ministry of Health should collaborate on policy development, while academic institutions should provide evidence-based educational materials. NGOs and community organisations can play a crucial role in grassroots outreach, particularly in coastal areas with fewer educational resources. Industry stakeholders, comprising seafood vendors, processors, and restaurant groups, ought to endorse these initiatives by providing explicit point-of-sale labelling regarding species origin, safety certificates, and preparation requirements.

Several factors influenced seafood safety and consumption knowledge, including the participants’ age, education level, family size, religion, and living in coastal areas. In contrast, seafood safety and consumption attitudes were shaped by education level, family size, employment status, presence of seafood allergies, and history of seafood poisoning. This study clearly revealed that highly educated people had greater knowledge and positive attitudes towards seafood safety and consumption than their uneducated or less educated counterparts. However, it is important to note that previous studies, such as those by Hicks *et al.* (2008) and Baptista *et al.* (2020), did not find a significant association between education level and seafood safety and consumption.^(29,32)^ This discrepancy may be attributed to differences in the study populations or contexts. This study was conducted on coastal populations in Bangladesh, where access to information about seafood safety and consumption is often limited, particularly in rural areas or communities with lower levels of education. In such settings, individuals with higher education levels may have better access to the media, health campaigns, and formal education, which can enhance their knowledge and attitudes towards seafood safety. In contrast, studies by Hicks *et al.* (2008) and Baptista *et al.* (2020) may have been conducted in regions with better access to seafood-related information, or their study populations may not have had the same level of disparity in education levels.^(29,32)^ Additionally, factors such as socioeconomic status, local food culture, and availability of seafood-related health education may differ between our study and theirs, contributing to varying outcomes.

Moreover, our study indicates that individuals residing in coastal areas have demonstrated higher knowledge regarding seafood safety and consumption than their non-coastal counterparts. This disparity is attributed to regular exposure to seafood as a dietary staple and better access to fresh seafood, which fosters familiarity with its health benefits and nutritional value. Living in proximity to marine resources enhances knowledge through cultural practices, frequent visits to seafood markets, and local insights into safe seafood consumption. These factors collectively explain the observed differences in seafood knowledge between coastal and non-coastal residents.

Our study identifies several key motivators that influence consumers’ decisions to purchase seafood. Freshness was the primary factor, followed by taste, health, safe to eat, locally sourced, price, and easy cooking. This aligns with the findings reported by Carreras-Simó *et al.* (2023) in Spain, where freshness, quality, taste, trust, and local sourcing were identified as significant drivers of seafood consumption.^(33)^ Similarly, Ali *et al.* (2010) reported that consumers prioritise freshness, followed by price, quality, packaging, and non-seasonal availability when purchasing products year-round when making purchasing decisions.^(34)^ These consistent findings across studies suggest that freshness is universally recognised as a crucial motivator influencing seafood consumption decisions. Trust in labelling further boosts consumer confidence in the freshness of seafood, as verified labels on origin, sustainability, and handling practices ensure quality. In terms of barriers to purchasing seafood, our study found key demotivators such as unavailability, high cost, lack of information about the availability of sustainable local seafood, personal preferences, and a lack of knowledge on how to prepare it. These findings are consistent with those of Murthy *et al.* 2023, who reported that high price, unknown flavour, did not know how to cook or prepare, unappetising aspects, and did not try unfamiliar products because the origin of the food was a significant deterrent to seafood consumption.^(35)^

Limited access to the market and inefficiency in supply chains contribute to disparities in seafood availability and affordability. In this study, unavailability and high prices were identified as significant barriers to seafood consumption. These challenges are exacerbated by cold chain infrastructure, logistical constraints, and market centralisation, which restrict access to fresh seafood, especially in remote or inland areas. Furthermore, price fluctuations resulting from mismatches between demand and supply affect affordability, making it difficult for lower-income consumers to purchase seafood regularly. Enhancing local supply chains, improving transportation networks, and investing in sustainable aquaculture can improve market accessibility and affordability. Structural improvements, such as upgrading the seafood market infrastructure, establishing hygienic processing facilities, and expanding cold chain logistics, can significantly boost seafood safety and accessibility.

In our study, the most commonly consumed seafood was rupchanda, followed by shrimp, coral fish, and loitta. These preferences reflect of the regional availability and cultural significance of certain species in the country. A separate study conducted among consumers in Dhaka City, Bangladesh, revealed that they preferred Ilish, Rupchanda, Shrimp, Loitta, Churi, and Tuna.^(36)^ Some foods, such as poa and phasa, were favoured because of their affordability. This highlights the importance of economic factors in shaping seafood preference. The most commonly consumed seafood in Canada includes salmon, tuna, shrimp, cod, and crab, whereas in the U.S., the top five species are shrimp, salmon, canned tuna, catfish/pangasius, and tilapia.^(37)^ These differences can be attributed to a range of factors including local fish availability, purchasing power, and the influence of international trade on seafood options. Furthermore, the prevalence of processed and imported seafood such as canned tuna and frozen shrimp is higher in these regions, further distinguishing their consumption patterns from those observed in Bangladesh.

The findings from our study reveal distinct patterns in seafood purchase and consumption compared with other studies. We observed that 40% of participants purchased seafood from local markets, which is lower than the 71% reported by Rahman *et al.* 2020.^(38)^ This difference may reflect variations in the availability of alternative purchasing options or in regional economic factors. Additionally, 62% of our participants indicated that seasonality did not influence their seafood consumption, in contrast to Wake Gelato (2019), who highlighted that fish demand is significantly affected by seasonal changes.^(39)^ This discrepancy could be due to regional variations in fish availability (because certain regions may have more stable seafood availability year-round), pricing, or cultural practices related to seasonal consumption. Although affordability was a major concern for the participants, seasonality was not perceived as a limiting factor. This paradox may be explained by the consistent availability of farmed seafood, preserved fish, or imports, which mitigate seasonal shortages. Additionally, consumer purchasing power and species preference may influence perceptions of affordability more than seasonal price fluctuations do. Moreover, the frequency of seafood consumption in our study showed that 21.2% of participants consumed seafood twice a week, whereas Hicks *et al.* (2008) found that 35% of Americans consumed seafood once a week or more, with a more polarised pattern of regular and infrequent consumption.^(32)^ These differences highlight the influence of geographical, cultural, and economic factors on seafood consumption across diverse populations.

Our study highlights the key environmental concerns that influence seafood consumption among consumers. The most alarming environmental dangers identified were overfishing, climate change, pollution and wastage of rivers and streams, loss of biodiversity, loss of animal species, and Illegal, Unreported, and Unregulated (IUU) fishing. These findings share some common themes with a global seafood consumer survey conducted in early 2020 by GlobeScan, an independent research and strategy consultancy.^(40)^ This survey, which included 26000 consumers from 23 countries, found that climate change, pollution and waste-damaging rivers and streams, extreme weather events, air pollution, destruction of rain forests, health of oceans, decline in fish populations (35%), and loss of animal species (32%) were the most concerning environmental threats to seafood consumers. While the specific rankings differ between the two studies, both highlight a significant awareness of environmental issues among seafood consumers. This awareness is likely driven by media coverage, public campaigns, and a growing sense of responsibility among consumers for making sustainable choices.

### Policy implications and regulatory context

The results of this study, including the crucial knowledge-attitude gap, preference for species such as rupchanda, and concerns about overfishing, must be examined within Bangladesh’s developing seafood control policy. Although the Marine Fisheries Act, 2020 and Fish and Fish Products (Inspection and Quality Control) Rules, 1997 provide fishing limits and quality criteria, their application does not help solve the discrepancies found in this study.^(41,42)^ For example, the urban-centric enforcement of the Safe Food Act, in 2013 exacerbated knowledge gaps in coastal communities, where informal markets dominate, despite higher seafood exposure.^(43)^ Similarly, export-oriented policies (e.g. shrimp aquaculture subsidies) increase preferences for commercial species while neglecting the biodiversity concerns raised by the participants. The persistence of price barriers and seasonal availability issues, despite the cold chain provisions in the 1998 National Fisheries Policy, highlights systemic supply chain failures. Most importantly, participants’ knowledge of IUU fishing and pollution contrasts significantly with the poor application of the Hilsa Conservation Rules and gear limitations. These regulatory gaps highlight the need for distributed, community-engaged strategies that link ground reality in coastal Bangladesh with legal frameworks (e.g. traceability requirements under Fish Rules, 1997). By addressing these gaps, regulations can be transformed from paper-based mandates to instruments that empower consumer knowledge, ensure equitable access, and protect marine ecosystems.

### Limitations

This study had several limitations that should be acknowledged. First, the use of convenience sampling may introduce selection bias, as the participants were selected based on accessibility rather than random sampling, which limits the generalizability of the findings to the broader coastal population. While the interviewers were trained, the potential for interviewer bias during face-to-face interviews could not be entirely ruled out. The cross-sectional design of the study restricts the ability to establish causality between sociodemographic factors and consumers’ knowledge, attitudes, and behaviours. Finally, the possibility of non-response bias and the limited exploration of cultural or regional factors may have influenced the results. These limitations should be considered when interpreting our findings.

## Conclusion

This study is the first to explore seafood safety knowledge, attitudes, preferences, and environmental concerns in coastal communities in Bangladesh. Our findings reveal a significant gap in seafood safety knowledge (48.2%), despite relatively positive attitudes (63.5%). Factors such as education, coastal residency, and personal seafood experiences shaped these patterns, with higher education levels being linked to better knowledge and attitudes. Freshness and taste emerged as key motivators, whereas price and unavailability were significant barriers. Environmental concerns such as overfishing and climate change were prevalent among respondents, reflecting a growing awareness of sustainability issues. Cultural preferences, economic factors, and local seafood availability also influence consumption patterns, with rupchanda being the most popular choice. Therefore, implementing targeted educational campaigns aimed at enhancing seafood safety knowledge, especially for those with lower levels of education, is essential to close the existing knowledge gap. Additionally, it is essential for local authorities to improve the availability of affordable and sustainable seafood choices, while also highlighting the associated health benefits. Future research should investigate the effectiveness of educational interventions in enhancing seafood safety knowledge and attitudes, especially among groups with lower educational backgrounds. Furthermore, longitudinal studies that examine environmental changes influence seafood consumption behaviours and safety concerns would offer valuable insights.
